# Anther development of maize (*Zea mays*) and longstamen rice (*Oryzalongistaminata*) revealed by cryo-SEM, with foci on locular dehydration and pollen arrangement

**DOI:** 10.1007/s00497-015-0257-3

**Published:** 2015-02-10

**Authors:** Chih-Hua Tsou, Ping-Chin Cheng, Chiung-Maan Tseng, Hsiao-Jung Yen, Yu-Lan Fu, Tien-Rong You, David B. Walden

**Affiliations:** 1 Institute of Plant and Microbial Biology, Academia Sinica, Taipei, Taiwan, ROC; 2 Department of Electrical Engineering, State University of New York, Buffalo, NY 14221 USA; 3 Chia-yi Branch, Tainan District Agricultural Improvement Station, Tainan, Taiwan, ROC; 4 Emeritus, Department of Biology, University of Western Ontario, London, ON N6A 5B7 Canada

**Keywords:** Central pollen, Cryo-SEM, Locular dehydration, Nutrient transportation, Peripheral pollen, Pollen arrangement

## Abstract

**Pollen maturation in Poaceae.**

Another development has been extensively examined by various imaging tools, including transmission electron microscopy, scanning electron microscopy, and light microscopy, but none is capable of identifying liquid water. Cryo-scanning electron microscopy with high-pressure rapid freeze fixation is excellent in preserving structures at cellular level and differentiating gas- versus liquid-filled space, but rarely used in anther study. We applied this technique to examine anther development of Poaceae because of its economic importance and unusual peripheral arrangement of pollen. Maize and longstamen rice were focused on. Here, we report for the first time that anthers of Poaceae lose the locular free liquid during late-microspore to early pollen stages; the majority of pollen grains arranged in a tight peripheral whorl develops normally and reaches maturity in the gas-filled loculus. Occasionally, pollen grains are found situated in the locular cavity, but they remain immature or become shrunk at anthesis. At pollen stage, microchannels and cytoplasmic strands are densely distributed in the entire pollen exine and intine, respectively, suggesting that nutrients are transported into the pollen from the entire surface. We propose that in Poaceae, the specialized peripheral arrangement of pollen grains is crucial for pollen maturation in the gas-filled loculus, which enables pollen achieving large surface contact area with the tapetum and neighboring grains to maintain sufficient nutrient flow. This report also shows that the single aperture of pollen in Poaceae usually faces the tapetum, but other orientation is also common; pollen grains with different aperture orientations show no morphological differences.

**Electronic supplementary material:**

The online version of this article (doi:10.1007/s00497-015-0257-3) contains supplementary material, which is available to authorized users.

## Introduction

 The anther in the flower is responsible for producing and releasing male gametophytes, namely pollen grains. Several extraordinary structural or conformational changes take place during anther development. (1) Each microspore mother cell (MMC) gives rise to four microspores within the callosic wall after meiosis, and then, these microspores become free cells after callose degradation (Scott et al. 2004). (2) A liquid-filled cavity called a loculus is created in each microsporangium after meiosis, and the developing microspores or pollen grains are immersed in the locular fluid for nutrient acquisition (Pacini et al. 2006; Pacini 2010). (3) The loculus becomes dehydrated before the anther opens, which allows the pollen grains to first adapt to a gaseous environment (Pacini et al. 2006; Pacini 2010). (4) Anther dehisces and pollen grains are released, which involves marked breakdown and stretching of the wall layers (Wilson et al. 2011).

Anther and pollen development have been intensively studied with conventional imaging tools such as transmission electron microscopy (TEM), scanning electron microscopy (SEM), and light microscopy (LM). Use of these tools has been informative, but the chemical fixation and/or dehydration required for sample preparation inevitably alters the cell dimensions (and morphology), so detailed detection of structural changes is difficult. In addition, none of these tools can distinguish the liquid- and gas-filled compartments. Confocal fluorescent microscopy and multiphoton fluorescent microscopy can be used to study living specimens, but a useful “tag” for water in the tissue is lacking, and the living anther shows high light-scattering properties and is too bulky for effective imaging (Cheng 2006).

In contrast, use of cryo-SEM with high-pressure rapid freeze fixation (HPRF–cryo-SEM), freeze fracture, and low temperature (−190 °C) observation is superior to the above-mentioned tools in understanding dynamic changes inside the anther loculus but has not been used extensively. Using HPRF–cryo-SEM, El-Ghazaly et al. (2000) and Rowley et al. (2003) reported certain types of strands extending from the tapetum to the pollen surface in anthers of *Magnolia* and *Betula*; the strands were proposed to function in nutrient transport, but information on other aspects was not provided.

Poaceae, including maize, wheat, rice, barley, and sorghum, are the most economically important family in the world. Studies of anther development of Poaceae are important for both basic botanical research and agronomic applications. Previous studies of the structural and cytological aspects of anther development have revealed important information for both maize (Greyson et al. 1980; Cheng et al. 1983, 1986; Chang and Neuffer 1989; Cheng and Pareddy 1994; Cheng 1997, Bedinger and Fowler 2009) and rice (Cheng et al. 1981; Raghavan 1988; Matsuo and Hoshikawa 1993; Wilson and Zhang 2009; Zhang et al. 2011).

Anthers of Poaceae and Cyperaceae are unique in that all microspores or pollen grains are arranged in one peripheral whorl throughout development, whereas in most angiosperms, pollen grains fill up the entire loculus (Kirpes et al. 1996; Pacini 2010). Also, pollen of Poaceae is characterized by having a single aperture and the pore always faces the tapetal side (Christensen and Horner 1974; Kirpes et al. 1996). Anthers of Poaceae usually have four wall layers: the middle layer degenerates the earliest, the glandular-type tapetum degenerates slowly during pollen development but retains a persistent tapetal orbicular wall (called the peritapetal layer in other reports) (El-Ghazaly and Jensen 1987), and the endothecium and the epidermis persist. At maturity, pollen in each microsporangium is enclosed directly by a tapetal orbicular wall, then the endothecium and the epidermis (Christensen et al. 1972; Cheng et al. 1979; Wilson and Zhang 2009), and then is covered by a continuous layer of cuticle (Cheng et al. 1986).

Microspores and pollen grains are free cells inside the loculus and rely on the locular fluid for nutrient supply (Clement and Audran 1999; Pacini 2010). The locular fluid is believed to disappear by re-absorption and/or evaporation before the anther opens and the maturing pollen grains undergo dehydration (Firon et al. 2012); such locular dehydration prepares the pollen grains for exposure in a gaseous atmosphere. However, direct data for the water content and movement in the anther loculus are scarce (Pacini 2010; Firon et al. 2012), and the timing and process of locular dehydration have never been documented in detail because use of conventional imaging tools has been inconclusive in differentiating air-space and liquid-space.

Here, we report the first HPRF–cryo-SEM study of anther development from early meiosis until anthesis in Poaceae; particularly, we examined the peripheral pollen arrangement and the locular dehydration of anthers. Our study of anther dehiscence will be presented in a separate report.

## Materials and methods

### Materials

Samples of Poaceae used included four inbred lines of maize (*Zea mays* subsp. *mays* L.) (B73, Gaspé, Ohio 43, W23), teosinte (*Zea mays* subsp. *parviglumis*), rice (*Oryza* *sativa* L.), longstamen rice (*Oryza longistaminata* A. Chev et Roehr.), barley (*Hordeum vulgare* L.) and a non-crop species, *Setaria viridis* (L.) P. Beauv. Maize (Ohio 43) and longstamen rice were examined throughout development because the size of anthers was more suitable for processing. The observations of the remaining species focused on microspore or pollen arrangement and locular dehydration. These samples represented four genera from three subfamilies (Ehrhartoideae, Panicoideae, and Pooideae) of the 12 subfamilies of Poaceae.

All the anther samples were taken from florets on the main rachis of the inflorescences; in maize with two florets in each spikelet, only the upper florets were used for consistency in developmental progress. Anthers representing 1 day before anthesis (1 DBA) were taken from the region slightly below the region of earliest flowers on the first day of anthesis. The sequences of anthesis of maize and rice are available in Hsu and Peterson (1981) and Hoshikawa (1993).

## Methods

For cryo-SEM study, florets were immediately dissected after removal from plants. For all the samples, one anther from each floret was fixed in 50 % EtOH or 2 % glutaraldehyde for further identification and the remaining anthers were placed onto sample stubs and rapidly frozen in the cryo chamber (Quorum PP2000TR) under −210 °C for at least 15 s. After the HPRF, the sample stub was transferred into the preparation chamber of a cryo-SEM system (Quanta 200, FEI), where the frozen anther was fractured (at −160 to −180 °C) and/or etched (sublime water) (25 min at −100 °C) and sputter gold-coated (2 min at −130 °C), then transferred to the observation chamber, and observed under a secondary electron emission mode at −190 °C.

Because of the size of the specimen and thermal conductivity of water, ice crystal formation may not be avoided in the deeper parts of the specimen, which would cause damage to the subcellular structures and create artifacts within the cells; the most common artifacts are reticulum and bars in the images (Figs. 2IB, IC, 3IF, 4IB, ID). Nevertheless, such small-scale ice crystal formation does not alter the morphological features at the cellular or tissue level, nor the identification of water in the sample. Etching is used to help the identification of water content, and gold coating is used to improve the secondary electron emission signals and minimize charging, thereby improving the image contrast for structural identification.

For confocal microscopical studies, anthers were immediately fixed in 50 % EtOH or 2 % glutaraldehyde, rinsed with 1 % phosphate buffer, stained with 1 % aniline blue in buffer overnight, rinsed with buffer, and examined under near UV excitation (405 nm) with a Zeiss LSM 510 Meta confocal microscope. For preparing the TEM images of the tapetal orbicular wall, which are provided in the Online Resource 2, the ZnCl_2_–HCL-lysis (acetolysis) method was used to reveal the sporopollenin-based portion in the tapetal orbicular wall, the preparation followed Cheng and Lin (1980). For preparing the TEM images of the exine and intine, which are provided in Online Resource 3, the procedure followed Tsou and Fu (2007).

## Results

### Anther development

**Fig. 1:**
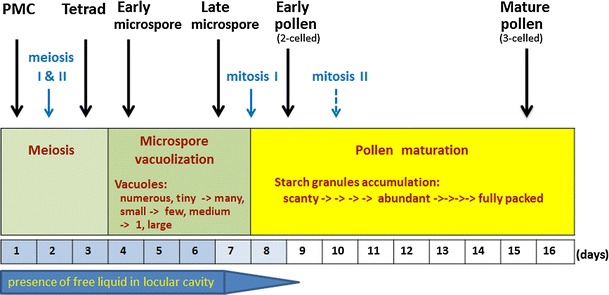
Time course of anther development of upper floret of maize (Ohio 43) starting from stage of microspore mother cell (MMC) to anthesis. Three stages are identified: meiosis, microspore, and pollen. During microspore stage, the progressive vacuolization is the most distinctive event. And the pollen stage (maturation phase) is characterized by the gradual accumulation of starch granules. The durations of these three stages can be variable; for example, pollen maturation in the inbred line Ohio 43 takes 7–10 days or slightly longer depending on the growth condition. The timing of mitosis II, indicated by a *dash line*, is based on limited samples and requires further studies

The upper florets of maize (Ohio 43) take about 14–17 days to develop from the stage of microspore mother cell (MMC) to anthesis. The meiotic stage from MMC to tetrad takes about 3 days, microspore stage 4 days, and pollen stage 7–10 days (Fig. 1).

#### Meiosis to tetrad stages

**Fig. 2:**
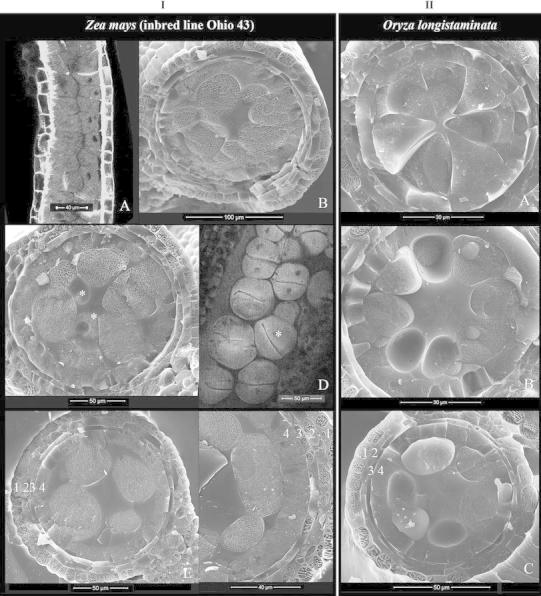
Anthers at stages of microspore mother cell (MMC), meiosis I, and meiosis II of maize (Ohio 43) (*Zea mays*) (**I**) and longstamen rice (*Oryza* *longistaminata*) (**II**). **I**A, **I**D are images of confocal microscopy of a longitudinal section (l.s.) of a microsporangium. Other figures are images of cryo-SEM of a cross section (c.s.) of a microsporangium. **I** Maize (Ohio 43). A and B, stage of MMC. MMCs are in square shape in l.s. and triangular in c.s.; 5–6 MMCs are arranged in one whorl in c.s. C, stage between MMC and meiosis I. MMCs change from *triangular* to *spherical shape,* and dyads are all adjacent to the tapetum; occasionally, dyads are located relatively away from the tapetum (*). D, stage of dyad. Dyads are *spherical*-*shaped*; the new cell plate is mostly perpendicular to the tapetal inner surface and less often obliquely oriented (*). The paired daughter cells are in an above-and-below arrangement. E and F, stage of meiosis II. Dyads stretch laterally in c.s. indicating the undergoing of a nuclear division. **II** Longstamen rice. A, stage of early MMC; 6–8 MMCs are arranged in one whorl and *triangular*-*shaped* in c.s. B, stages of MMC and early meiosis I. MMCs change from *triangular* to *spherical*. C, stage of meiosis II. Dyads change to *oblong*-*shaped* in c.s. In both species, a locular central cavity is created and filled with liquid; four wall layers are well present: epidermis (1), endothecium (2), middle layer (3), and tapetum (4)

**Fig. 3:**
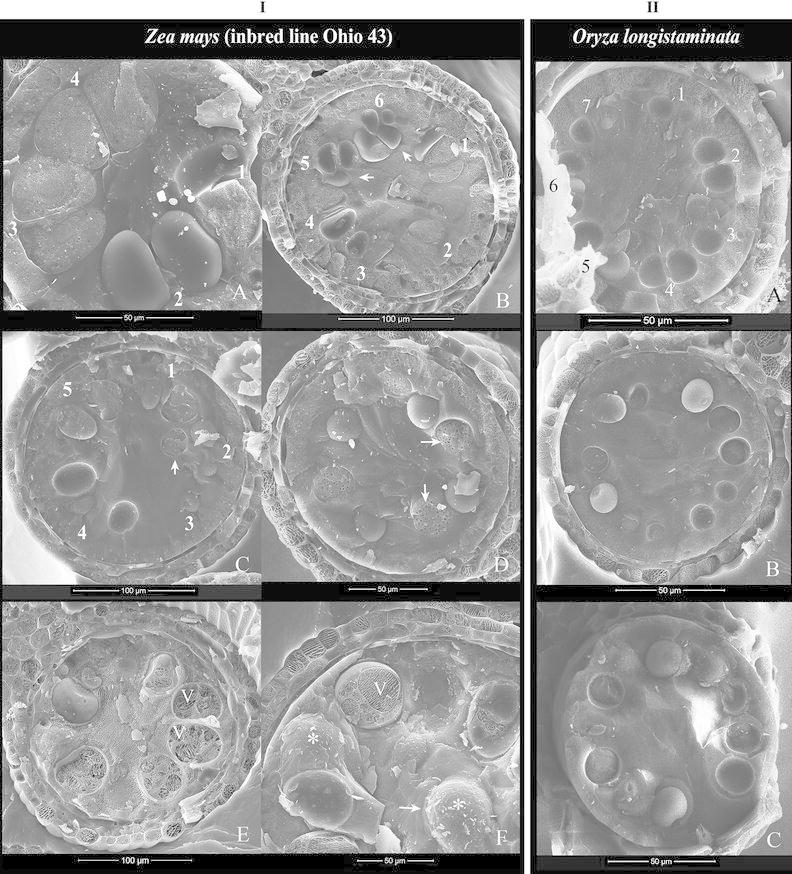
Cryo-SEM images of anther c.s. at stages from tetrad to mid-vacuolated microspore of maize (Ohio 43) (**I**) and longstamen rice (**II**). **I** Maize (Ohio 43). A and B, stage of tetrad. Four and six tetrads as numerically labeled are present in A and B, respectively. Usually, only one pair of microspores from a tetrad can be seen in c.s., such as the pairs in tetrads 2, 3, and 4 in A. If tetrads are obliquely oriented (*arrows* in B), one or two microspores are relatively away from the tapetum. C and D, stage of early free microspore. Five tetrads are numerically labeled in C. Microspores are loosen from the pairing and become *elliptical*-*shaped* and evenly distributed in one whorl. Occasionally, microspores located in locular cavity can be seen (*arrow* in C). Soon, very small vacuoles start to form (*arrows* in D). E and F, stage of mid-vacuolated microspore. Microspores contain several medium-sized vacuoles (V), they contact each other laterally and the tapetum distally, yet they remain elliptical-shaped in c. s. A developing exine with a rough surface can be seen (*in F). **II** Longstamen rice. A, stage of tetrad. Seven tetrads as labeled with 1–7 can be seen. Microspores are in pairs, with a flat inner radial wall due to the mutual compression as in tetrads 2, 4, and 7. B, stage of early free microspore. Microspores are *spherical* in shape, separated, and evenly distributed in a peripheral whorl. C, stage of mid-vacuolated microspore. Microspores contact each other laterally, yet they remain *spherical* in shape. In both species, the locular central cavity is filled with liquid

In maize (Ohio 43) and longstamen rice, each microsporangium had four to six and five to eight MMCs, respectively, arranged in one whorl in a cross section. The early MMCs were rectangular-shaped in a longitudinal section (Fig. 2IA) and triangular-shaped in a cross section (Fig. 2IB, IIA). The deposition of callose was mainly in the center of the loculus and MMCs adhered to the tapetum (Fig. 2IB). The cytokinesis of MMCs was of the successive type, and MMCs changed from a triangular to spherical shape during meiosis I (Fig. 2IC, ID, IE, IIB). The cell plate of cytokinesis I was mostly horizontal and perpendicular to the inner tapetal surface (Fig. 2ID). The dyads would stretch laterally to become elliptical-shaped during meiosis II (Fig. 2IF, IIC), and cell plates of cytokinesis II were perpendicular to the cell plate I and to the inner tapetal surface, which resulted in tetragonal tetrads with the four microspores equally close to the tapetum. Usually, only two microspores of a pair from each tetrad could be seen in the cross section (Fig. 3IA, IIA). Occasionally, the cell plates of cytokineses I and II were not perpendicular to the inner tapetal surface (Fig. 2IC, ID), which resulted in obliquely oriented tetrads, with one or even two of the four microspores positioned away from the tapetum (Fig. 3IB). At early tetrad stage, the pairing of microspores was easily recognized based on the flat inner radial wall caused by the mutual compression and the arched outer radial wall (Fig. 3IA, IIA). By the end of the tetrad stage, the anther wall consisted of four cellular layers, with the epidermis and tapetum much thicker than the endothecium and middle layer (Fig. 3IB, IIA).

#### Callose degradation and microspore stages

After cytokinesis II, the callosic walls started to degrade, which was judged indirectly by the progressive changes in the spacing and shape of young microspores. First, the microspores remained in pairs but became slightly separated and attained an elliptical shape in maize (Ohio 43) (Fig. 3IC) and more or less spherical in longstamen rice because of the decreased mutual compression. Then, all the microspores became evenly spaced in the peripheral whorl, and the pairing was no longer recognizable (Fig. 3ID, IIB). During the early stage of free microspore, these young microspores stayed separated and had no visible contact with each other or with the tapetum (Fig. 3IC, ID, IIB); nevertheless, they seemed to be well fixed in the peripheral position based on several criteria: (1) Their long axis was always in a radial direction; (2) they were equally close to the tapetum and evenly spaced in the whorl (Fig. 3ID, IIB); and (3) they seemed not to float freely. Occasionally, microspores were found outside the whorl and relatively far away from the tapetum (Fig. 3IC).

**Fig. 4:**
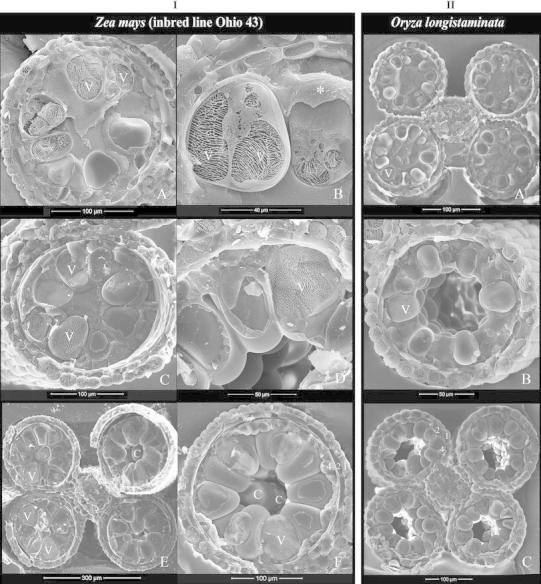
Cryo-SEM images showing the process of locular dehydration and anther features at stages from late-vacuolated microspore to early pollen of maize (Ohio 43) (**I**) and longstamen rice (**II**). **I** Maize (Ohio 43). A and B, stages of mid- to late-vacuolated microspore. Microspores have only few vacuoles (V), become *wedge*-*shaped,* and keep large surface contact with each other laterally. Exine surface is rough (*in B). C, stage of late-vacuolated microspore. Microspores contain a single large vacuole (V), become *wedge-shaped*, and tightly compress with each other and the tapetum. The locular cavity is filled with liquid. D and E, stages from late-vacuolated microspore to early pollen. Locular dehydration proceeds from the center of the cavity toward the periphery; the four microsporangia of an anther may not dehydrate in synchrony. F, stage of early pollen. The loculus has no free liquid. Pollen grains contain a single large vacuole (V) and have a smooth exine surface. Central grains (C) can be seen in the locular cavity. The tapetum (4) is of varying thickness because of compression from pollen. **II** Longstamen rice. A, stage of late-vacuolated microspore. The locular cavity is filled with liquid. Microspores contain a single large vacuole (V). B and C, stages of late-vacuolated microspore and early pollen. Locular dehydration takes place from central cavity toward the periphery. Pollen grains become *wedge-shaped* and have large surface contact with each other laterally. In both species, three wall layers are well present: epidermis (1), endothecium (2), and tapetum (4), with the epidermis the thickest

During the microspore stages, microspores underwent active vacuolization. At first, vacuoles were numerous but tiny (Fig. 3ID). Later on, several small-sized vacuoles were present, and the microspores of maize (Ohio 43) were elliptical and that of longstamen rice were spherical. They came in slight contact with each other laterally and with the tapetum distally; a complete whorl of microspores was built up (Fig. 3IE, IF, IIC). At mid-vacuolated stage, there were few medium-sized vacuoles present; microspores of maize (Ohio 43) became oval- to wedge-shaped, with a broad distal side and a narrower proximal side, whereas those of longstamen rice became oblong-shaped. By the late-vacuolated stage, each microspore had a single large vacuole; microspores of both species were wedge-shaped (i.e., with several flat lateral sides, a broad distal end and a narrow proximal end) (Fig. 4IC, IIA).

The anther loculus was filled with liquid throughout microspore stages (Figs. 2, 3, 4IA, IC, IIA). Sometimes microspores were found in the central cavity of the loculus, which was more common in maize (Oh43) than longstamen rice. These central grains were basically spherical in both species. Therefore, the wedge shape of the peripheral microspores seemed to be resulted from compression from each other. As for the development of exine, a developing exine could not be detected at very early microspore stage (Fig. 3ID, IIB), but was seen at early- and mid-vacuolated microspore stages, though the layer was not well defined and had a rough surface (Figs. 3IE, IF, IIC, 4IB). Only at late-vacuolated stage did the exine appear as a distinct layer with a clean and smooth surface (Fig. 4IC, IIA).

#### Dehydration of anther loculus at late-microspore and early pollen stage

During the transition from late-vacuolated microspore to early pollen stage, a prominent event took place, whereby the anther loculus became dehydrated (Fig. 4ID–IF, IIB, IIC). The center of the loculus lost free liquid first and a hollow column was created (Fig. 4IIB); then, free liquid was lost from the peripheral cavities between the tapetum and the microspores (Fig. 4ID). The dehydration in the loculus was not synchronized among the four microsporangia of an anther (Fig. 4IE). Since the early pollen stage, the anther loculus was gas-filled. Without locular fluid, the interior of the anther was clear on SEM: The majority of pollen grains were tightly packed in a peripheral whorl and remained wedge-shaped (Fig. 4IF, IIC); some central grains dispersed in the central cavity were found in both maize (Ohio 43) and longstamen rice; central grains were spherical and smaller than peripheral grains (Fig. 4IE, IF); pollen exine had a clean surface covered with uniformly spaced fine granules, a typical ornamentation of Poaceae pollen, but the exine layer was easily torn apart during the freeze–fracture process (Fig. 4IF, IIC).

By the early pollen stage, the anther wall of both species was composed of three layers: epidermis, endothecium, and tapetum. The epidermis was thicker than the endothecium (14 vs. 8 μm in maize [Ohio 43]; 10.5 vs. 4.5 μm in longstamen rice). The tapetal layer thickness varied a lot because of the uneven compression from the peripheral grains (Fig. 4IF, IIC), and on the inner surface of the tapetum, a layer of tapetal orbicular wall was developed.

**Fig. 5:**
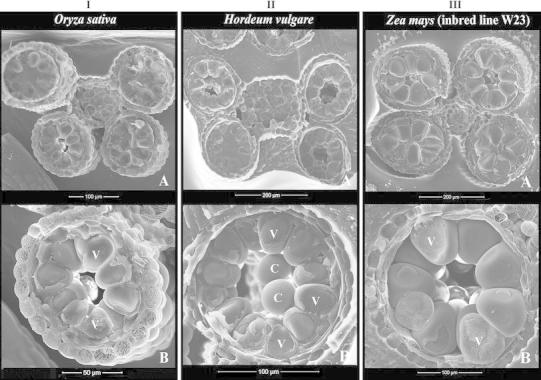
Cryo-SEM images showing the process of locular dehydration and anther features at stages from late-vacuolated microspore to early pollen of rice (*Oryza* *sativa*) (**I**), barley (*Hordeum vulgare*) (**II**), and maize (W 23) (**III**). In all these species, locular dehydration proceeds from the center of the cavity toward the periphery and the four microsporangia of an anther may not dehydrate in synchrony. Pollen grains are *wedge*-*shaped*, with large surface contact with each other laterally and a single large vacuole (V) (**I**B, **II**B, **III**B). Central grains (C) can be seen in the locular cavity; they are *spherical*-*shaped* (**II**B). Three wall layers are well present: epidermis, endothecium, and tapetum (**I**B, **II**B, **III**B). The epidermis is especially well developed in rice (**I**B)

In addition to maize (Ohio 43) and longstamen rice, the other samples all showed locular dehydration at late-microspore and early pollen stages: rice (*Oryza sativa*) (Fig. 5IA, IB), barley (*Hordeum vulgare*) (Fig. 5IIA, IIB), maize inbred lines W23 (Fig. 5IIIA, IIIB), B73 and Gaspé (not shown), teosinte (*Zea mays* subsp. *parviglumis*) (not shown), and *Setaria* *viridis* (not shown). Also, the locular dehydration proceeded from the center of the loculus toward the periphery and was not synchronized among the four microsporangia of an anther in these species (Fig. 5IA, IIA, IIIA). In addition to locular dehydration, many other features were uniformly expressed in all these samples at the early pollen stage: pollen grains arranged in a peripheral whorl, occasional presence of central grains, peripheral grains in wedge shape, central grains in spherical shape, and the features of the wall layers (epidermis thicker than endothecium, tapetum of varying thickness), etc. (Fig. 5).

#### Pollen maturation

**Fig. 6:**
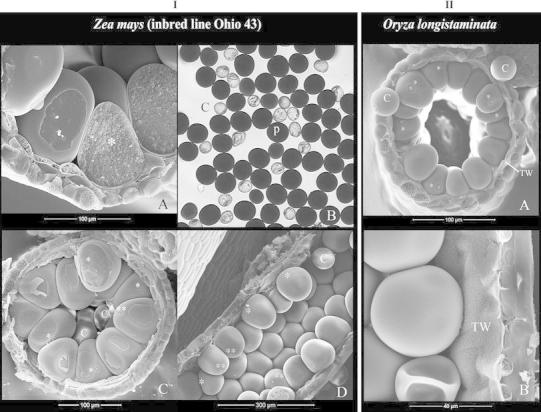
Cryo-SEM images of anthers and pollen grains at mature stage of maize (Ohio 43) (**I**) and longstamen rice (**II**) except that **I**B is viewed under a light-transmitted microscope. **I** Maize (Ohio 43). A, mature pollen grains are filled with starch granules and exhibit a dense cytoplasm (*). They are *wedge*-*shaped*, with large surface contact with each other laterally and with the anther wall distally. B, at maturity, peripheral grains (P) are large and appear dark because of high content of light-scattering starch granules; the central grains (C) are smaller and appear transparent because of lack of starch granules. C and D, mature anthers in c.s. and l.s. Please note the *wedge-shaped* peripheral grains tightly compressed to the anther wall and the presence of smaller and *spherical* central grains (C). Some peripheral grains have the aperture facing laterally (*) or toward the central cavity (**). **II** Longstamen rice. Mature pollen grains are tightly arranged in a peripheral whorl and compressed to the anther wall; free central grains (C) are smaller and *spherical*. The tapetum is degraded, with a tapetal orbicular wall (TW) remained, which is densely covered with orbicules. Pollen aperture facing laterally (*) is common as seen in A. In both species, mature anther wall includes a predominant epidermis, a thin endothecium, and a tapetal orbicular wall

Starting from early pollen stage until the beginning of anthesis, pollen grains steadily accumulated starch granules (Fig. 6IA). During the entire phase of pollen maturation, which took about 7–10 days in maize (Ohio 43), the anther loculus was devoid of free liquid; the peripheral wedge-shaped grains were tightly compressed laterally and distally (Fig. 6IA, IIA) and enlarged from 55–65 to 95–100 μm long in maize (Ohio 43) and from ca. 25 to 40–45 μm in longstamen rice. Mature peripheral grains were filled with starch granules and appeared as dark grains on bright-field light microscopy (Fig. 6IB). The pollen exine surface was clean and rather smooth (Fig. 6IC, ID, IIA, IIB). The tightly packed peripheral arrangement and all of the above-mentioned pollen features remained to anthesis.

During pollen maturation, the central grains remained turgid or became shrunk. They were found in all species examined, and their frequency varied among the inbred lines of maize: fairly common in Ohio 43 but less common in other lines. These central grains were not well anchored physically, and thus, they would pop out from the central cavity and land on other parts of the sample when the anther was freeze-fractured (Fig. 6IIA), but they could be easily distinguished from the peripheral grains. They were spherical, smaller than the peripheral grains (ca. 50–70 μm in diameter in maize [Ohio 43] and 30–35 μm in longstamen rice) (Fig. 6IC, ID, IIA), lack of starch granules, and transparent on bright-field light microscopy (Fig. 6IB). These central grains were mostly viable as tested by FDA fluorescence (data not shown), but they were highly vacuolated and shrank immediately when released from the anther; therefore, they should be considered immature and abnormal grains.

At maturity, the tapetal layer was already much degraded; only the tapetal orbicular wall remained, which was covered with orbicules (Ubisch bodies) densely (Fig. 6IIA, IIB). Pollen grains in each microsporangium were enclosed by this tapetal orbicular wall and then surrounded by a poorly developed endothecium and a predominant epidermis (Fig. 6IA, ID, IIA, IIB). The above-mentioned characteristics such as the peripheral arrangement of pollen, the morphological features of peripheral pollen and central pollen, and the anther wall composition were also observed in barley, rice, other maize inbred lines (B73, Gaspé, W23) [Online Resource 1], teosinte, and *Setaria* *viridis*.

#### Pollen aperture orientation

Pollen of Poaceae is characterized by having a single aperture. In samples of all maize inbred lines, rice, and barley, the aperture of the peripheral pollen was mostly oriented toward the tapetum when observed; less frequently, peripheral pollen showed the aperture facing a lateral side or the central cavity (Fig. 6IC, ID) [Online Resource 1]. However, in longstamen rice, the aperture facing the lateral side was very common (Fig. 6IIA) [Online Resource 1]. In all samples, the peripheral grains with the aperture facing the lateral side or the tapetal side did not show any distinctive differences morphologically (Fig. 6IC, ID, IIA) [Online Resource 1].

## Discussion

In this HPRF–cryo-SEM study, anther samples were well preserved and showed minimal distortions at the cellular level; the gas-filled and liquid-filled spaces were well retained. We revealed many features not known previously and information complementary to and contradictory to our current understanding. In the discussion, we focus on how and why the unusual peripheral pollen arrangement is established in the Poaceae and the possible routes of nutrient supply when pollen develops in the gas-filled loculus. It is intended to integrate our understanding of the pollen distribution in Poaceae from both physical and physiological perspectives.

### Peripheral arrangement of microspore/pollen, consistency, and mechanical basis

In most angiosperms, microspores disperse freely in the anther loculus when released from the callosic wall, and then, they expand via vacuolization and take up the entire space of the loculus. In Poaceae and Cyperaceae, however, microspores and pollen grains are organized into a single peripheral whorl in the loculus, with a large central cavity free of pollen (Kirpes et al. 1996; Pacini 2010). How such peripheral arrangement is established and maintained is of interest. Christensen and Horner (1974) explained the mechanical mechanisms of two developmental stages in Poaceae. First, at the meiotic stage, various polarities exist: the callose mainly deposited at the center, meiocytes situated at the tapetal side, and the spindles of meiosis I and II always parallel to the tapetal inner surface, which cause the four meiotic microspores situated at the periphery and equally adjacent to the tapetum. The control mechanisms of the fixed orientation of meiotic spindles were demonstrated later (Staiger and Cande 1990). Second, at the vacuolated microspore stage and afterward, microspores become compressed against each other laterally and to the tapetum distally; this mutual compression acts as the retaining force holding the grains in the peripheral whorl. However, the status of the microspores during the transition between these two stages (i.e., the early free microspore stage) was not explained.

Our observations support the above-proposed mechanisms. We also observed that during the transitory period, the early free microspores were always well anchored in position and never randomly dispersed. When tetrads were just produced, the paired microspores were enclosed by the callosic walls and each had an arched outer radial wall and a flat inner radial wall in cross sections (Fig. 3IA, IIA). Then, the degradation of callosic wall occurred, as indicated by the loosening of the paired microspores and the inner radial wall becoming arched because of reduced mutual compression; finally, all microspores became evenly distributed in the peripheral whorl (Fig. 3IC, ID, IIB). During this transitory phase, the microspores were always aligned in the radial direction, evenly distributed in the peripheral whorl, not in contact with each other, and maintained an approximate equal distance from the tapetum. Such separation yet well-organized distribution pattern suggests that these microspores are well anchored in the loculus. This arrangement persisted until the microspores expanded enough to compress against each other laterally at the early- to mid-vacuolated stage (Fig. 3IE, IF, IIC). The same pattern was observed in barley and other maize inbred lines. Apparently, the early free microspores of Poaceae are “free of contact” but do not float freely; they seem to be anchored at their position. The mechanism providing such physical constraints is not clear and not determined in this study, but callose might be involved. Callose in the center of the tetrad retains the microspore association for some time during the early microspore stage in *Sorghum* (Christensen et al. 1972), is not digested completely and participates in holding the microspores together to form compound pollen in *Annona* and *Cymbopetalum* (Annonaceae) (Tsou and Fu 2002, 2007), and performs different functions in various conditions, such as creating a scaffold or microenvironment for deposition of wall materials (Cheng and Lin 1980; Galatis and Apostolakos 2010). In Poaceae, the callose digestion may proceed slowly, so that microspores are released from the callosic wall steadily and do not float around during the “free-of-contact” period; later, they build up enough turgor pressure to form a complete whorl, and then, the mutual compression acts as the retaining force.

We also noticed that once the microspores were arranged in a peripheral whorl during the mid-vacuolated stage, the arrangement became tighter gradually until early anthesis (Figs. 4IE, IIC, 5IB, IIB, IIIB, 6IA, IC, IIA) [Online Resource 1]. The gradual increase of the mutual pressure and the pressure toward the tapetum were observed in all the samples studied. Structurally, the wedge-shaped pollen grains packed tightly in a circular ring resemble stones in an arched bridge; so, the force in the arched structure stabilizes the entire structure as well as the individual grains. Theoretically, it is difficult to rationalize whether the microspores are randomly distributed first and then organized into a peripheral whorl in the anthers of Poaceae. Therefore, images in many previous reports of Poaceae showing spherical-shaped and well-separated peripheral grains or random distribution of microspores or pollen, etc., could be artifacts. Grass anther material is difficult to fix consistently well (Christensen and Horner 1974), and the integrity of the tight whorl of pollen grains can be easily destroyed by decreasing the turgor pressure in just a few grains during preparation for conventional TEM, SEM, and LM studies. Also, an earlier report of the breakdown of anther septum in maize at least 24 h before anthesis (Keijzer et al. 1996) is questioned because septal region is vulnerable and can be artificially broken down easily.

### Central pollen

Although Poaceae is characterized by exhibiting peripheral arrangement of pollen grains in the anther, central grains dispersed in the loculus cavity were found in all species examined (Figs. 4IE, IF, 5IIB, 6IC, ID, IIA) [Online Resource 1]. The central grains are often viable at anthesis but remain highly vacuolated and immature; therefore, they are abnormal grains. As mentioned above, various preconditions and mechanisms exist in establishing and maintaining the peripheral arrangement of pollen in Poaceae. So, how are these central grains produced? Some clues could be deduced from the meiotic stages in both maize (Ohio 43) and longstamen rice. After meiosis I, the cell plate I was found not perpendicular to the tapetal inner wall occasionally, which resulted in obliquely oriented dyads (Fig. 2IC, ID) and, furthermore, obliquely orientated tetrads, in which one or two microspores in the tetrad would be relatively away from the tapetum (Fig. 3IB). When callosic walls were being degraded, all the microspores expanded slowly at their original sites; those microspores relatively far away from the tapetum were then not wedged in properly or were even pushed away from the whorl of microspores (Fig. 3IC); then, these grains may only engage in the tightly packed whorl partially or be expelled into the central cavity. These “orphan” microspores would become the central grains. The peripheral space of the loculus not sufficient to accommodate the expansion of all the MMCs or the events of meiosis I and II not proceeding normally is assumed to produce obliquely oriented dyads/tetrads. In *Triticum*, the isobilateral tetrad is predominant, yet other types with imbalanced distribution of microspores do occur (Deshpande and Raju 1985). From the view of structural physics, once a continuous whorl of microspores is formed, the mutual pressure starts to increase gradually; either inserting a grain into the whorl or removing one from the whorl seems no longer possible in normal condition. The presence of the abnormal central grains in Poaceae is rather common but has not been clearly documented. In our experience, under unfavorable growing conditions, the proportion of these abnormal grains would increase. The peripheral grains and the central grains are not believed to differ in their nature; their localities within the loculus (i.e., with direct contact with the tapetum or not) determine their fates. The correlation between physical and physiological factors is discussed below.

### Dehydration of anther loculus and nutrient supply for pollen maturation

In anthers with a secretary type of tapetum, such as Poaceae, the developing microspores or pollen are believed to obtain nutrients from the locular fluid, which contains secretions from the tapetum and serves as a transitory site for sugar storage (Castro and Clement 2007; Pacini 2010). However, reports of the quantity and quality of the locular fluid are limited (Pacini and Franchi 1983; Clement et al. 1998; Pacini et al. 2006; Castro and Clement 2007; Pacini 2010; Firon et al. 2012), mainly because of the difficulties in extraction for analysis. Also, the locular fluid disappears via evaporation and/or reabsorption before anther dehiscence (Pacini 2000; Firon et al. 2012), and thus, pollen is ready for dispersal when the anther opens. However, the timing and process of locular dehydration have never been clearly demonstrated because of limitations in imaging tools.

Regarding the routes of nutrient uptake into pollen cytoplasm, various sites, including pollen aperture, microchannels or irregular channels in the exine, and exine bulges, were demonstrated via tracer experiments (Fernandez and Garcia 1990; Rowley et al. 1987; 2003). In addition, an extracellular system of strands between the tapetal surface and pollen surface found in *Poa* (Rowley 1962) and *Caltha* (Cheng and Lin 1980) on TEM and in *Magnolia* and *Betula* on cryo-SEM (El-Ghazaly et al. 2000; Rowley et al. 2003) was suggested as a route for nutrient transport (Rowley et al. 2003). In this study, we detected no extracellular system on the pollen surface, but we observed that the anther loculus became dehydrated at the early pollen stage in all the species studied (Figs. 1, 4ID, IIB, 5IA, IIA, IIIA) and even the loculus was gas-filled throughout the maturation phase (ca. 7–10 days in B73 and Ohio 43 of maize); peripheral pollen always developed into mature grains normally; and the central pollen mostly remained viable but immature (Figs. 5IB, IIB, IIIB, 6IC, IIA) [Online Resource 1]. Such early dehydration in the loculus has never been reported in Poaceae or other angiosperms, as far as we know. Because the locular dehydration takes place when the vacuolization in the microspore approaches the maximum, the free fluid may be taken up by the microspores. Then, how the poaceous pollen grows normally and reaches maturity in the gas-filled loculus needs to be addressed.

In view of the contrast of the locality and the physiological status between the peripheral and central grains, many clues can be deduced: The peripheral grains oppress to the anther wall so tightly that they retain a large surface contact with the anther wall layers and are able to accumulate enormous amounts of starch granules; they expand from ca. 65 to 100 μm during the maturation phase and reach maturity normally. However, the central pollen grains are in near-isolation and only contact the peripheral grains tangentially but not the anther wall at all; they accumulate limited starch granules and remain immature. Most of the available nutrients provided by the anther wall layers are assumed to be taken up by the peripheral grains, and the central grains experience a significantly lower nutrient gradient than the peripheral grains. It is assumed that a layer of boundary liquid may form a continuum from the inner surface of the anther wall (i.e., the tapetal orbicular wall) to the surface of peripheral grains and then to the surface of central grains. This layer of boundary liquid would envelop the entire pollen grain and the nutrient transport into the cytoplasm be conducted via the whole pollen surface.

Such a transportation pathway in Poaceae is well supported. First, peripheral grains constantly retain a large surface contact with the tapetal orbicular wall (i.e., the anther wall). Second, the tapetal orbicular wall itself is not a solid layer of sporopollenin; it is porous and the orbicules on this wall also contain many microchannels (Christensen et al. 1972; Cheng 1997) [Online Resource 2]. Third, the exine (including sexine and nexine) possesses densely distributed microchannels, as seen in maize (Skvarla and Larson 1966), *Poa* (Rowley 1962), *Sorghum* (Christensen et al. 1972), *Triticum* (El-Ghazaly and Jensen 1985, 1987), and our own data of maize and rice [Figs. B, D, E, F of Online Resource 3]. Fourth, during the phase of pollen maturation, the inner pollen wall, intine, is built up beneath the exine; densely distributed cytoplasmic strands are developed in radial direction in the intine, as seen in *Poa* (Rowley 1962), *Sorghum* (Christensen et al. 1972; Christensen and Horner 1974), *Triticum* (El-Ghazaly and Jensen 1986, 1987), and our own samples of maize and rice [Figs. D, E, F of Online Resource 3], which implies that nutrients are actively transported into the cytoplasm via the entire pollen wall. Fifth, the microchannels in the pollen exine and those in the tapetal orbicules are of similar diameter and can be connected (El-Ghazaly and Jensen 1987).

We propose that microspores and pollen grains of Poaceae are nourished by different pathways at different developmental stages. During early free microspore until mid-vacuolated microspore stages, the tapetum is well present, the tapetal orbicular wall is not well developed, and microspore exine is not well built up. Microspores are immersed in the locular fluid and take up nutrients mainly from the free locular liquid. At late-vacuolated microspore and early pollen stages, pollen grains become firmly compressed against the tapetum and neighboring grains. The pollen exine and the tapetal orbicular wall are now well built up; although they are made of impermeable sporopollenin, the numerous microchannels and pores inside these two structures provide substantial passages. Meanwhile, the loculus loses free liquid and the tapetum starts to degenerate during pollen development [Online Resource 3]. A new transport pathway is established that nutrients from the anther wall layers pass through the porous tapetal orbicular wall, move within the layer of boundary liquid, reach the surface of peripheral grains and further the surface of central grains, and then travel through the microchannels in exine and the cytoplasmic strands in the intine to reach the pollen cytoplasm [Online Resource 3]. This nutrient transportation pathway seems the main route during the entire phase of pollen maturation because of the lack of free locular fluid. During this phase, pollen acts as nutrient sink and pollen of Poaceae actively takes up a large amount of sucrose for starch synthesis (Clement and Audran 1999), which is supplied by the photosynthetic tissues. Meanwhile, the much-diminished tapetum apparently is not important in the sucrose supply. The peripheral arrangement of pollen in Poaceae is crucial for pollen maturation because only those pollen grains retaining a large surface contact with the tapetum or anther wall are able to acquire sufficient nutrients and reach maturity in the gas-filled loculus. However, why the early dehydration of anther loculus is evolved in Poaceae is unknown.

### Pollen aperture orientation

The single aperture of poaceous pollen is reported to be oriented toward the tapetal side and suggested to function in nutrient transportation (Christensen and Horner 1974; Kirpes et al. 1996). In all species we examined, the peripheral grains mostly had the aperture facing the tapetum, yet a small proportion had the aperture facing lateral side or even the central cavity (Fig. 6IC, ID) [Online Resource 1]; longstamen rice is an exception, in which the aperture of peripheral grain facing a lateral side was fairly common (Fig. 6IIA) [Online Resource 1]. And, we found no morphological differences between grains with the aperture facing the tapetum and those with the aperture facing laterally in the same pollen chamber. Pollen aperture is the site for pollen tube germination and may perform other functions, such as nutrient and water uptake and accommodating the volume change. In Poaceae, the entire pollen exine possesses densely distributed microchannels, including the cap and the thickened margin of the pollen aperture (El-Ghazaly and Jensen 1986), which all may serve as nutrient passages.

## Conclusions

Poaceae and Cyperaceae have a peripheral arrangement of pollen grains unique among angiosperms, but this specialized pattern could not be explained. It is generally believed that in anthers with a secretory type of tapetum, as in Poaceae, pollen grains are immersed in and absorb nutrients directly from the locular fluid, and the loculus becomes dry out before anthesis, but the locular liquid and the locular dehydration had not been clearly demonstrated previously. In this study, HPRF–cryo-SEM is used to examine the anther development of Poaceae for the first time. It shows that the locular cavity starts to dehydrate at the stages of late-vacuolated microspore and early pollen; nevertheless, pollen grains which are retained in a compact peripheral whorl and tightly oppress to the tapetum develop into mature grains in the gas-filled loculus normally. The study suggests that in Poaceae, the peripheral arrangement of pollen grains is a prerequisite for pollen maturation. Such a physiological success relies on proper physical coordination, including the well-programmed meiotic events to place all microspores situated equally adjacent to the tapetum, the maximum number of well-positioned microspores to build up a compact whorl, and the compactness to keep pollen grains having a large surface contact with the tapetum or anther wall to acquire sufficient nutrients. Pollen grains failing to be allocated within the peripheral whorl will become central grains and remain immature. This cryo-SEM study also demonstrates that the single aperture of the Poaceae pollen is not necessarily facing the tapetum and the orientation of the aperture causes no obvious effect on pollen development.

### **Author contribution statement**

CH Tsou, PC Cheng, DB Walden initiated project & designed experiments; PC Cheng, CM Tseng, HJ Yen, YL Fu, TR You, DB Walden grew plants & conducted experiments. CH Tsou, PC Cheng and DB Walden wrote the manuscript.

## Electronic supplementary material

Below is the link to the electronic supplementary material.

1. Cryo-SEM images showing anther and pollen features and the orientation of pollen aperture in mature anthers of three inbred lines of maize (*Zea mays*), longstamen rice (*Oryza longistaminata*), and rice (*Oryza sativa*). A. Maize (B73). B. Maize (Gaspé). C. Maize (W23). D. Longstamen rice. E and F. Rice. At maturity, peripheral grains are *wedge-shaped* with large surface contact with each other laterally and with the anther wall distally; they are heavily packed with starch granules (St) (Fig. A). The central grain (C) is *spherical* in shape and much smaller than the peripheral grains (Figs. A, B, C, and F). The aperture of pollen if observable is marked (*) in these six figures; it mostly orients toward the tapetal side, but in longstamen rice it often faces laterally (Fig. D). The anther wall layers include a prominent epidermis, a thin endothecium, and a tapetal orbicular wall (TW) (Figs. A, D) (JPEG 2870 kb)

2. TEM images showing acetolyzed tapetal orbicular walls in maize (Ohio 43). The anther wall was treated with ZnCl_2_-HCL-lysis method (acetolysis), and the sporopollenin-based portion of the tapetal orbicular wall remained because sporopollenin is resistant to acetolysis. The images show that the tapetal orbicular wall is not a solid layer of sporopollenin. A. Lower magnification showing the tapetal orbicular wall attached with several pollen grains (P). The sporopollenin-based portion of the wall is made of irregular-shaped reticula and orbicules. B. Higher magnification showing a few reticula and numerous fine orbicules (tiny dark dots). Each reticulum (one marked with white line) represents the margin of one tapetal cell at the proximal side. C. Higher magnification showing densely distributed orbicules (*) and orbicular aggregations (**) and cavities (JPEG 1852 kb)

3. TEM images showing the possible routes of nutrient transport in anther wall and pollen wall in anthers of maize (Ohio 43) (Figs. A-D) and rice (Figs. E–F). A-D. Maize (Ohio 43). A-B. Stage of late-vacuolated microspore. A. Microspores are tightly pressed to each other and onto the tapetum. Tapetum is of varying thickness due to the pressure from the microspores. B. Higher magnification showing the exine of microspore and the tapetal wall. Microchannels (black arrows) are well developed in both sexine and nexine of the exine. Tapetum is thin, and the tapetal orbicular wall is well developed and covered with orbicules. C-D. Late but not yet matured pollen. C. Peripheral pollen grains contain numerous, but are not yet fully packed with, starch granules. Tapetum is much compressed. D. Tapetum is completely compressed, but the tapetal orbicular wall remains. Numerous microchannels (black arrows) are present in the sexine and nexine of exine and numerous cytoplasmic strands (white arrows) in the intine. E-F. Late pollen of rice. Pollen grains contain large amount of starch granules. Microchannels (black arrows) are densely present in the sexine and nexine of exine and cytoplasmic strands (white arrows) in the intine. The lateral sides of two neighboring grains are shown in E and the distal side of one grain in F. The tapetal orbicular wall is well developed and densely covered with orbicules. Ed: endothecium; Ep: epidermis; I: intine, M: middle layer; N: nexine; O: orbicule; S: sexine; St: starch granules; T: tapetum; TW: tapetal orbicular wall (JPEG 3485 kb)
