# Supplementary figures and images for: Anther development of maize (Zea mays) and longstamen rice (Oryzalongistaminata) revealed by cryo-SEM, with foci on locular dehydration and pollen arrangement
Source: Plant Reprod. 2015 Feb 10;28(1):47–60. doi: 10.1007/s00497-015-0257-3 (PMC4333360; doi:10.1007/s00497-015-0257-3)

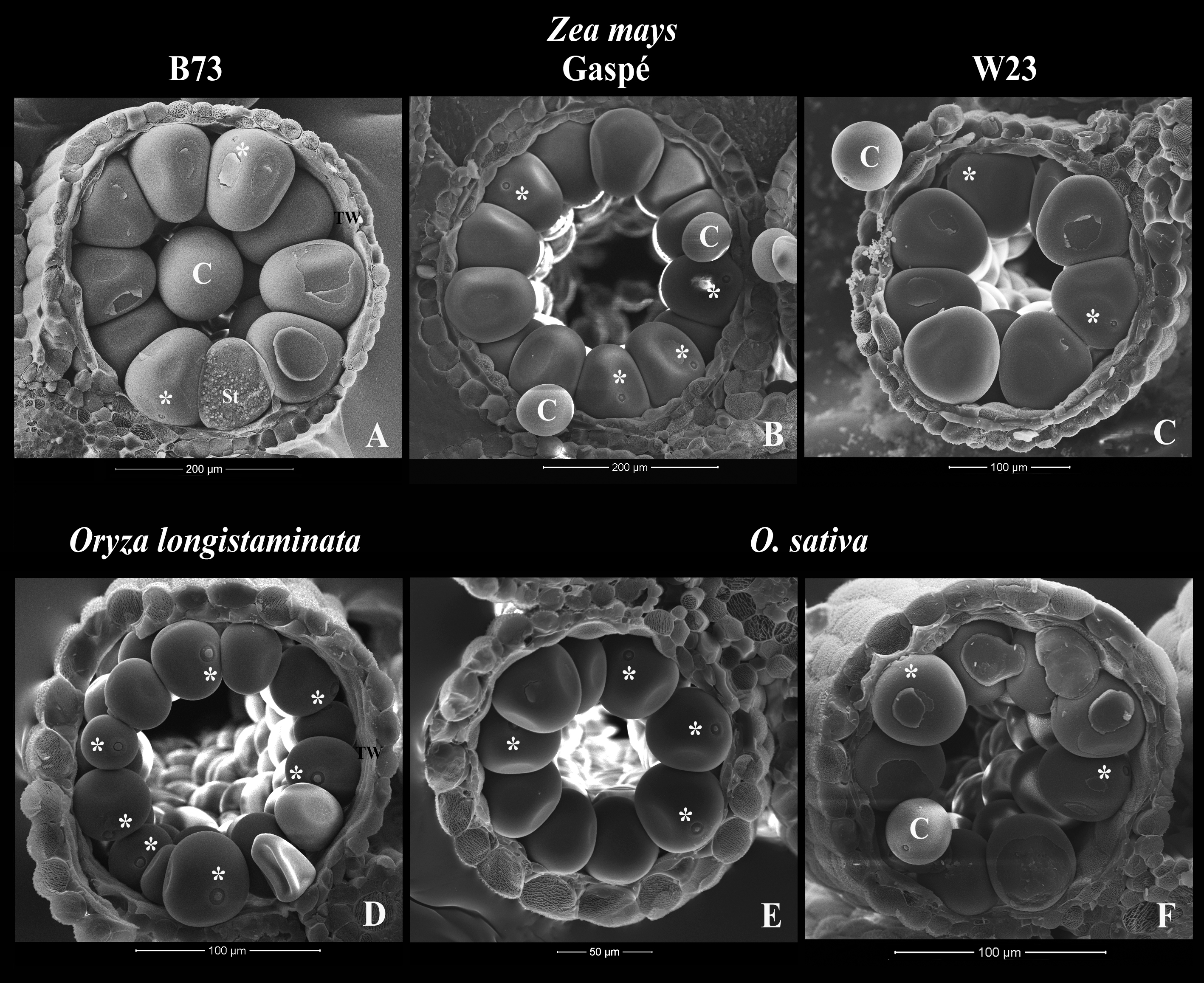

Supplement: Supplementary file 1 — 1. Cryo-SEM images showing anther and pollen features and the orientation of pollen aperture in mature anthers of three inbred lines of maize (Zea mays), longstamen rice (Oryza longistaminata), and rice (Oryza sativa). A. Maize (B73). B. Maize (Gaspé). C. Maize (W23). D. Longstamen rice. E and F. Rice. At maturity, peripheral grains are wedge-shaped with large surface contact with each other laterally and with the anther wall distally; they are heavily packed with starch granules (St) (Fig. A). The central grain (C) is spherical in shape and much smaller than the peripheral grains (Figs. A, B, C, and F). The aperture of pollen if observable is marked (*) in these six figures; it mostly orients toward the tapetal side, but in longstamen rice it often faces laterally (Fig. D). The anther wall layers include a prominent epidermis, a thin endothecium, and a tapetal orbicular wall (TW) (Figs. A, D) (JPEG 2870 kb) [file 497_2015_257_MOESM1_ESM.jpg]

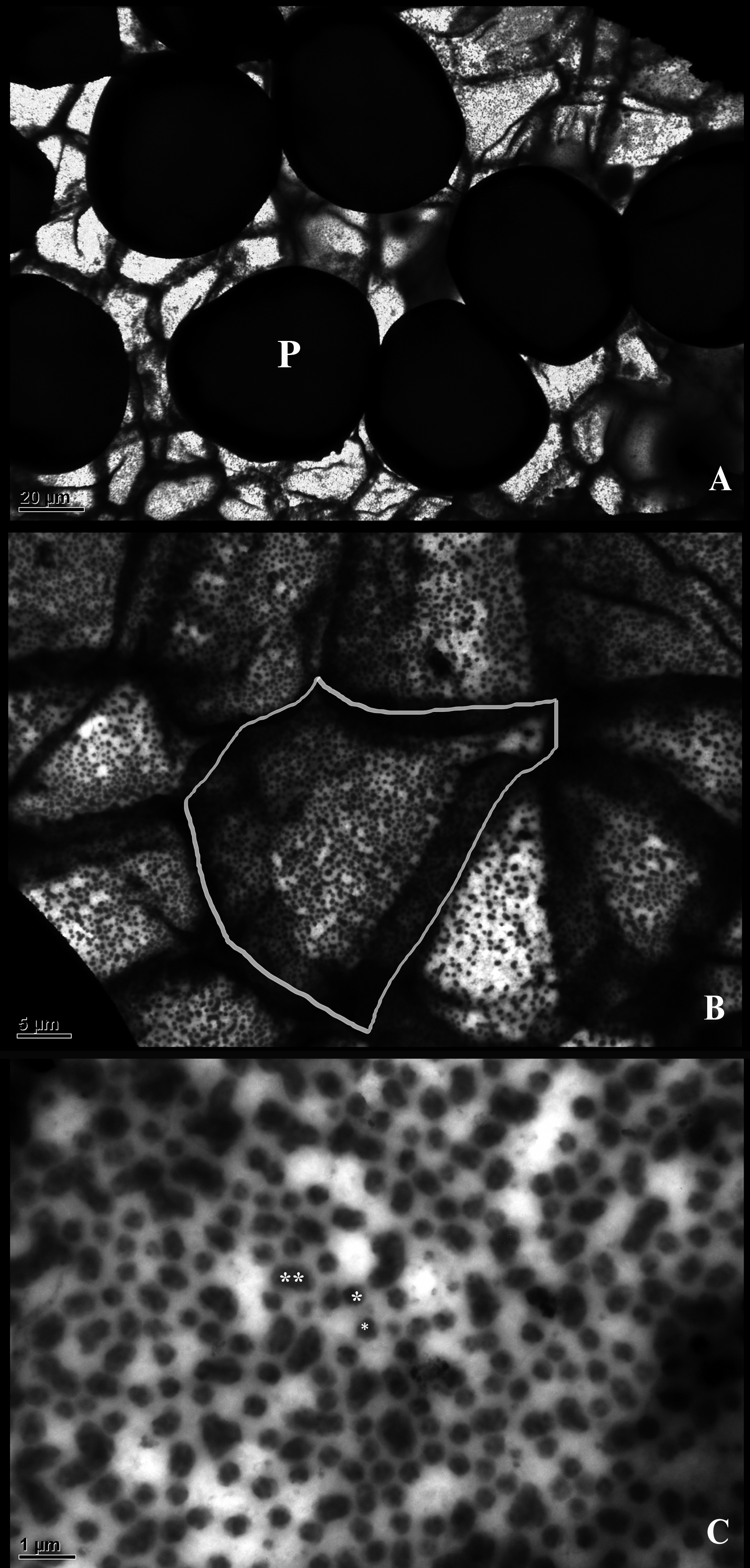

Supplement: Supplementary file 2 — 2. TEM images showing acetolyzed tapetal orbicular walls in maize (Ohio 43). The anther wall was treated with ZnCl2-HCL-lysis method (acetolysis), and the sporopollenin-based portion of the tapetal orbicular wall remained because sporopollenin is resistant to acetolysis. The images show that the tapetal orbicular wall is not a solid layer of sporopollenin. A. Lower magnification showing the tapetal orbicular wall attached with several pollen grains (P). The sporopollenin-based portion of the wall is made of irregular-shaped reticula and orbicules. B. Higher magnification showing a few reticula and numerous fine orbicules (tiny dark dots). Each reticulum (one marked with white line) represents the margin of one tapetal cell at the proximal side. C. Higher magnification showing densely distributed orbicules (*) and orbicular aggregations (**) and cavities (JPEG 1852 kb) [file 497_2015_257_MOESM2_ESM.jpg]

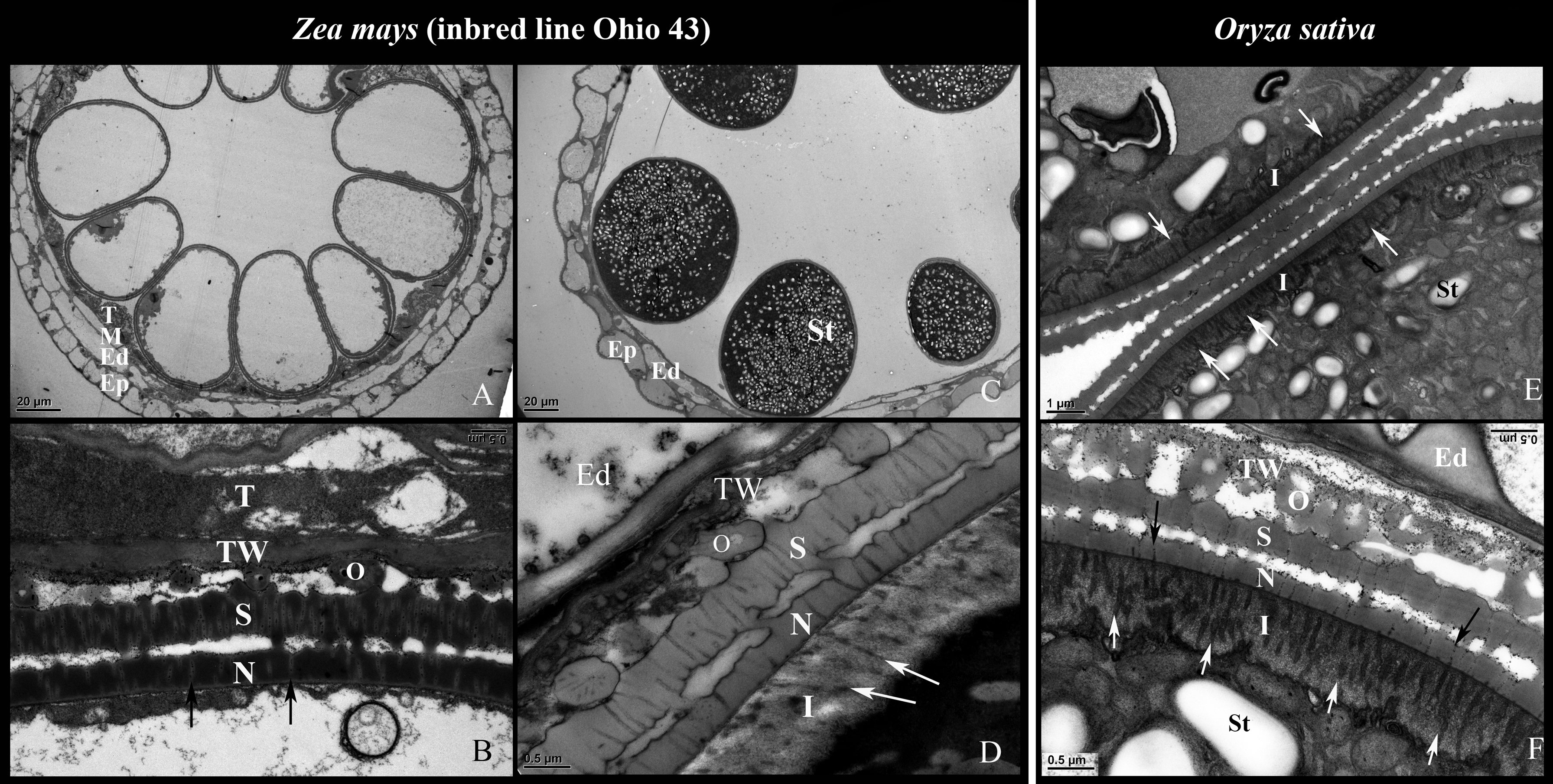

Supplement: Supplementary file 3 — 3. TEM images showing the possible routes of nutrient transport in anther wall and pollen wall in anthers of maize (Ohio 43) (Figs. A-D) and rice (Figs. E–F). A-D. Maize (Ohio 43). A-B. Stage of late-vacuolated microspore. A. Microspores are tightly pressed to each other and onto the tapetum. Tapetum is of varying thickness due to the pressure from the microspores. B. Higher magnification showing the exine of microspore and the tapetal wall. Microchannels (black arrows) are well developed in both sexine and nexine of the exine. Tapetum is thin, and the tapetal orbicular wall is well developed and covered with orbicules. C-D. Late but not yet matured pollen. C. Peripheral pollen grains contain numerous, but are not yet fully packed with, starch granules. Tapetum is much compressed. D. Tapetum is completely compressed, but the tapetal orbicular wall remains. Numerous microchannels (black arrows) are present in the sexine and nexine of exine and numerous cytoplasmic strands (white arrows) in the intine. E-F. Late pollen of rice. Pollen grains contain large amount of starch granules. Microchannels (black arrows) are densely present in the sexine and nexine of exine and cytoplasmic strands (white arrows) in the intine. The lateral sides of two neighboring grains are shown in E and the distal side of one grain in F. The tapetal orbicular wall is well developed and densely covered with orbicules. Ed: endothecium; Ep: epidermis; I: intine, M: middle layer; N: nexine; O: orbicule; S: sexine; St: starch granules; T: tapetum; TW: tapetal orbicular wall (JPEG 3485 kb) [file 497_2015_257_MOESM3_ESM.jpg]
